# Daytime naps and depression risk: A meta-analysis of observational studies

**DOI:** 10.3389/fpsyg.2022.1051128

**Published:** 2022-12-15

**Authors:** Liqing Li, Qi Zhang, Liyong Zhu, Guohua Zeng, Hongwei Huang, Jian Zhuge, Xiaorui Kuang, Sule Yang, Di Yang, Zhensheng Chen, Yong Gan, Zuxun Lu, Chunmei Wu

**Affiliations:** ^1^Research Center of Health Policy and Innovation, Jiangxi Science and Technology Normal University, Nanchang, Jiangxi, China; ^2^School of Public Health, Tongji Medical College, Huazhong University of Science and Technology, Wuhan, Hubei, China; ^3^School of Economics and Management, Jiangxi University of Science and Technology, Ganzhou, Jiangxi, China; ^4^Department of Health Management Medicine, The Second Affiliated Hospital of Nanchang University, Nanchang, China; ^5^School of Public Health and Health Management, Gannan Medical University, Ganzhou, Jiangxi, China

**Keywords:** daytime nap, depression, meta-analysis, psychiatry, mental health

## Abstract

**Background:**

The relationship between daytime napping and depression remains debatable. Thus, a meta-analysis in this study was conducted to evaluate the relationship between daytime napping and depression.

**Methods:**

The PubMed, Embase, Web of Science, and China National Knowledge Infrastructure databases were searched up to February 2022, and the reference lists of the included studies were also retrieved. A random-effects model was used to estimate the combined effect size.

**Results:**

Nine studies with 649,111 participants were included in the final analysis. The pooled odds ratio (OR) was 1.15 (95% confidence interval: 1.01–1.31) with a significant heterogeneity (*I*^2^ = 91.3%, *P* for heterogeneity <0.001), and the results demonstrated an increased risk of depressive symptoms among daytime nappers. Visual inspection of the funnel plot and Egger's and Begg's tests identified no obvious evidence of publication bias.

**Conclusion:**

This meta-analysis indicates that daytime naps are a predictor of depression. The effects of daytime napping on depression may vary depending on the characteristics of people, the pattern of naps, and the individual's sleep experience. The findings may have significant implications for future research on depression.

## Background

Depression is a common and disabling psychiatric condition worldwide (Dong et al., 2022; GBD 2019 Mental Disorders Collaborators, 2022), and it is a syndrome consisting of a variety of symptoms (such as depressed mood and loss of interest) (Izaki, 2021). The etiology of depression is complex, resulting from interactions between biological vulnerabilities and environmental factors (Alsaad et al., 2022). According to data from the Global Burden of Disease Study 2019, depressive disorders (major depressive disorder and dysthymia) were the leading causes of health-related issues worldwide, with a higher prevalence than many other diseases (GBD 2019 Mental Disorders Collaborators, 2022). The World Health Organization (WHO) has estimated that depression is expected to become the third cause of the global disease burden in 2030 (Malhi and Mann, 2018). This tendency would accelerate if risk factors were not identified and controlled effectively, both in natural and socioeconomic environments. For example, an additional 53.2 million cases of major depressive disorder globally (an increase of 27.6%) were estimated by the WHO due to the COVID-19 pandemic in the first year of the epidemic (COVID-19 Mental Disorders Collaborators, 2021). As one of the most widespread diseases affecting mental, physical, and social wellbeing, depression is receiving increasing attention.

Sleep is fundamental to a person's emotional and physical health (Patel et al., 2016; Dong et al., 2022). Many studies suggested that a series of sleep problems, including obstructive sleep apnea, circadian disorder, insomnia, and excessive daytime sleepiness, are associated with the risk of depression (Tsuno et al., 2005; Yu et al., 2016; Bixler et al., 2017). A daytime nap is a short sleep during daylight hours, and it is a habitual behavior in many regions around the world, such as Asia, Central America, and the Mediterranean region. In some countries, especially those with a nap culture, daytime napping is often considered one of the health-promoting lifestyle behaviors for adults, not only older people but also healthy adults and even athletes (Milner and Cote, 2009; Fang et al., 2013; Lastella M, 2021). Some studies showed that short naps are beneficial to increase alertness (Gillberg et al., 1996; Brooks and Lack, 2006; Faraut et al., 2011), motor skills (Hayashi et al., 1999; Sugawara et al., 2018), physical performance (Boukhris et al., 2020, 2022; Souabni et al., 2021), and cognitive performance (Asplund, 1996; Tamaki et al., 1999; Picarsic et al., 2008; Boukhris et al., 2020; Lastella M, 2021). Conversely, other researchers noted that daytime napping is associated with an increased risk of obesity (Wang et al., 2020), chronic diseases (Xu et al., 2010; Guo et al., 2017), and all-cause mortality (Leng et al., 2014b; Liu et al., 2015). Some studies suggested that the effect of daytime napping was related to its characteristics, especially the duration and frequency. Yamada et al. (2015) discovered that long daytime napping (≥60 min/day) could cause sleep inertia and was associated with a higher risk of cardiovascular disease and all-cause mortality. A dose-response meta-analysis in 2020 showed that the risk of stroke increased by 3% for every 10-min increase in daytime napping (Jin et al., 2021). Häusler et al. (2019) found that people who napped one time or two times weekly had a lower risk of cardiovascular disease incidents. A meta-analysis based on experimental studies in working-aged adults showed that cognitive performance increased after napping and that timing rather than duration influenced cognitive performance (Dutheil et al., 2021). Based on scientific evidence, the benefits of napping on psychomotor performance and learning abilities in older adults have also been discovered (Souabni et al., 2022). Cognitive impairment is an important symptom and outcome indicator of depression. Choi et al. (2020) found that daytime napping appeared to increase the risk of depressive symptoms. On the contrary, the study by Xie et al. (2020) suggested that daytime napping was a protective factor for depression. In recent years, more attention has been paid to the relationship between napping and cognitive and mental function.

The biological mechanisms neither between depression and daytime napping nor between daytime napping and the risk of depression were clear. Furthermore, a growing body of evidence shows contradictory results on the relationship between napping and depression. It is not clear whether napping is beneficial, detrimental, or neutral to our mental health (Vitiello, 2008). To date, a meta-analysis assessing the association between daytime napping and the risk of depression has not yet been conducted. Thus, we conducted a meta-analysis to investigate the effects of daytime napping on depression.

## Methods

### Literature search strategy

This meta-analysis was conducted in accordance with the guidelines of the Meta-analysis of Observational Studies in Epidemiology (MOOSE) (Stroup et al., 2000) and the preferred reporting items for systematic review and meta-analysis (PRISMA) statement (Moher et al., 2009). We systematically searched the PubMed, Embase, Web of Science (WOS), and China National Knowledge Infrastructure (CNKI) databases from February 2022 for studies describing an association between daytime napping and the risk of depression. We used the following keywords as search terms: “snooze,” “siesta,” “daytime napping,” “noontime nap,” “midday nap,” “noontime snooze,” “noontime nap,” in combination with “depression” or “depressive symptom.” In addition, all reference articles listed were reviewed. The search was conducted without language restrictions.

### Inclusion and exclusion criteria

Studies that fulfilled the following criteria were included: (1) the exposure of interest was daytime napping; (2) the outcome of interest was clinical or non-clinical depression, regardless of methods for diagnosis and severity assessment; (3) the study provided risk estimates such as relative risks (RR) or odds ratios (OR) with corresponding 95% confidence intervals (CIs) or sufficient data to calculate them; and (4) observational studies were included, i.e., cohort studies, cross-sectional studies, and case-control studies.

Studies were excluded if they were (1) not full reports; (2) duplicate studies; (3) animal studies; (4) studies on excessive daytime sleepiness rather than daytime napping; and (5) lacking adequate information to calculate risk estimates. Two reviewers (Q.Z. and D.Y.) independently screened and reviewed all studies by title, abstract, and full text. Disagreements were resolved through consultation with the third reviewer (C.M.W.).

### Data extraction

The following information for each included study was extracted: the first author's name, publication year, country, study design, follow-up year (only for cohort studies), age range or mean age of the participants (at baseline for cohort studies), gender, sample size, daytime napping definition and measurement, depression definition and measurement, adjusted covariates, and effect estimates with their corresponding 95% CI. Data extraction was conducted independently by two authors (K.J.Z. and Q.Z.). Interobserver agreement was assessed using Cohen kappa (κ), and any disagreements were resolved by discussion with the third author (Z.X.L.).

### Quality assessment

The methodological quality of the included studies was independently assessed by two reviewers (L.Q.L. and G.H.Z.) with appropriate tools. We used the Newcastle–Ottawa Scale (NOS) (Wells et al., 2019) to assess the quality of cohort and case-control studies. The NOS includes eight items grouped into selection, comparability, and outcome. Each study was assigned a score of 0–9. A NOS score of more than six indicated relatively high quality, of 5–6 indicated medium quality, and of <5 indicated low quality. The Agency for Healthcare Research and Quality (AHRQ) methodology checklist was applied to evaluate cross-sectional studies. The AHRQ checklist includes 11 items. We assigned scores as follows: 0–3 = low quality, 4–7 = moderate quality, and 8–11 = high quality.

### Statistical analysis

The OR value was considered the common measure of the association between daytime naps and depression. The multivariable-adjusted ORs were preferentially pooled when such estimates were reported. If the adjusted analysis was unavailable, the unadjusted estimates were pooled. A fixed-effect model was applied when heterogeneity was not detected. Otherwise, a random-effects model was used to summarize ORs for the association between daytime napping and depression. For further confirmation and assessment of the association between daytime napping and the risk of depression and the origin of heterogeneity, subgroup analysis was carried out to explore potential heterogeneity sources and examine the primary results' robustness. The differences among subgroups were tested by meta-regression analysis (using STATA's “metareg” command). Statistical heterogeneity among studies was evaluated with the *I*^2^ statistics, where values of 25, 50, and 75% represented cutoff points for low, moderate, and high degrees of heterogeneity, respectively (Higgins and Thompson, 2002). A sensitivity analysis was carried out by removing one study at a time to assess the source of heterogeneity and the magnitude of influence on the pooled OR of each study (Wallace et al., 2009). Potential publication bias was evaluated with a funnel plot, Begg's test (Begg and Mazumdar, 1994), and Egger's test (Egger et al., 1997). STATA software (v12.0, StataCorp, College Station, USA) was used to conduct the statistical analysis for this meta-analysis. All tests were two-sided, and a probability value < 0.05 was considered statistically significant.

## Results

### Literature search

**Figure 1:**
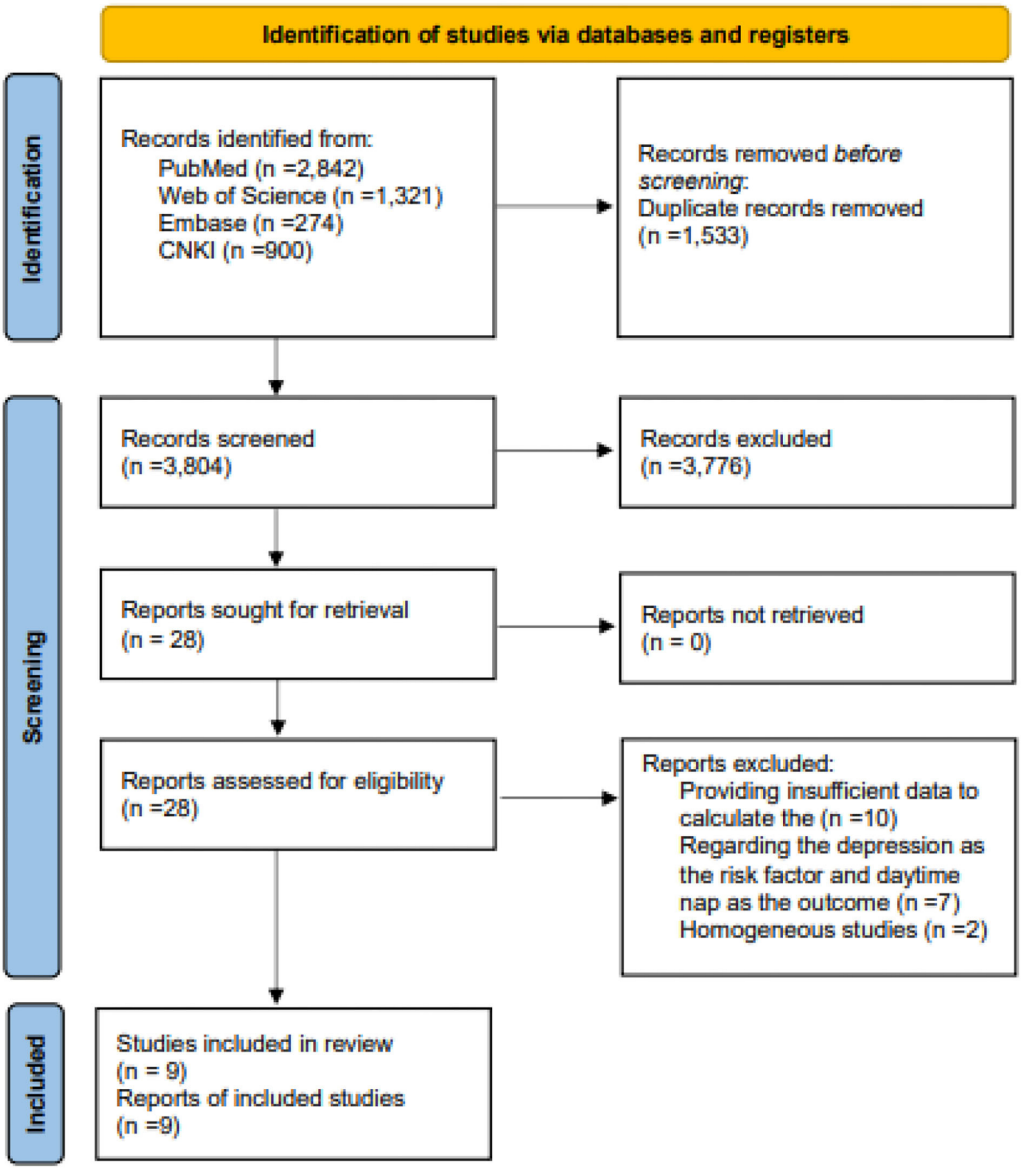
Flow chart of study selection.

The study screening process on the PubMed, Embase, WOS, and CNKI databases and the reference lists of the included studies retrieved a total of 5,337 studies. After eliminating duplicate publications and screening titles and abstracts, 28 articles were considered. At the full-text review stage, nine studies were eventually included in the meta-analysis. All steps and the reasons for exclusion are shown in Figure 1.

### Study characteristics

The characteristics of included nine studies (Foley et al., 2007; Leblanc et al., 2015; Liu et al., 2018; Ruiz-Estigarribia et al., 2019; Choi et al., 2020; Jing et al., 2020; Simoes Maria et al., 2020; Xie et al., 2020; Lin et al., 2021) are shown in Table 1. Our studies included 649,111 individuals, and the sample size of these studies ranged from 1,497 (Jing et al., 2020) to 512,891 (Liu et al., 2018). Most of the included studies (Leblanc et al., 2015; Liu et al., 2018; Ruiz-Estigarribia et al., 2019; Choi et al., 2020; Jing et al., 2020; Simoes Maria et al., 2020; Xie et al., 2020; Lin et al., 2021) were published after 2014, and only one study (Foley et al., 2007) was published before 2014 (in 2007). Four (Ruiz-Estigarribia et al., 2019; Choi et al., 2020; Jing et al., 2020; Lin et al., 2021) were cohort studies, and five (Foley et al., 2007; Leblanc et al., 2015; Liu et al., 2018; Simoes Maria et al., 2020; Xie et al., 2020) were cross-sectional studies. Of these included cohort studies, the lengths of follow-up years ranged from 1 year (Jing et al., 2020; Lin et al., 2021) to 17 years (Ruiz-Estigarribia et al., 2019), and the sample sizes ranged from 3,075 (Lin et al., 2021) to 118,373 (Choi et al., 2020). Four studies (Liu et al., 2018; Jing et al., 2020; Xie et al., 2020; Lin et al., 2021) were conducted in China, two studies (Foley et al., 2007; Leblanc et al., 2015) in North America, and three studies (Ruiz-Estigarribia et al., 2019; Choi et al., 2020; Simoes Maria et al., 2020) in Europe. All included studies (Foley et al., 2007; Leblanc et al., 2015; Liu et al., 2018; Ruiz-Estigarribia et al., 2019; Choi et al., 2020; Jing et al., 2020; Simoes Maria et al., 2020; Xie et al., 2020; Lin et al., 2021) provided only self-reported daytime napping, and only two of the included studies (Liu et al., 2018; Xie et al., 2020) reported results for men and women separately. A variety of depression diagnoses and assessments were used: four studies reported depressive symptoms using different self-reported questionnaires/scales (Choi et al., 2020; Xie et al., 2020; Lin et al., 2021), and five studies reported clinical depression by self-reported depression diagnosis (Foley et al., 2007; Ruiz-Estigarribia et al., 2019; Simoes Maria et al., 2020) or by health professionals (Leblanc et al., 2015; Liu et al., 2018). Self-reported nap information was collected in all nine included studies with different kinds of assessments. The results of the quality assessment are shown in Tables 2, 3. According to the NOS or AHRQ scores, all studies were of moderate or high quality.

**Table 1:** Characteristics of included studies in the meta-analysis.

**References**	**Study name**	**Sex**	**Country**	**Daytime napping measurement**	**Daytime** ** napping definition**	**Depression definition**	**Depression measurement**	**Study design**	**Age (years)**	**No of participants**	**Covariates**
Foley et al. (2007)	National Sleep Foundation's “2003 Sleep in America” Poll	M/F	US	Telephone interview	A self-reported nap 4–7 times a week	depression	Self-reported depression diagnosis with concomitant depressive symptom treatment	Cross-sectional	Range 55–84	1,479	Age and gender
Leblanc et al. (2015)	Quebec Survey on Seniors' Health (Enquête sur la santé des aînés)	M/F	Canada	Self-reported questionnaire structured interview	A self-reported nap during the day sometimes, often, or very often in the past month	A major depression, a minor depression, or a mania	Similar questions to those in the Diagnostic Interview Schedule	Cross-sectional	Mean age 73.8	2,759	Sex, age, marital state, income, and schooling
Liu et al. (2018)	China Kadoorie biobank	M/F	China	Self-reported questionnaire	A self-reported usual daytime nap	Major depression	Major depression defined by CIDI-SF	Cross-sectional	Mean 51.53 ± 10.65; Range 30–79	512,891	Residency, age, family mental disorder history, blood pressure, education, income, occupation, BMI, marital status, smoking, alcohol, MET statuses, sleep snoring, taking medicine for sleep, daytime dysfunction, difficulty falling asleep, and interrupted sleep, total sleep duration, and disease statuses
Ruiz-Estigarribia et al. (2019)	SUN	M/F	Spanish	HLS	A self-reported short afternoon nap (0.1–0.5 h/d)	depression	Self-reported depression diagnosis or habitual use of antidepressants	Cohort follow-up time: 2–17 years	Mean 36.7 ± 11.7	14,908	Sex, age, year of completion of the questionnaire, regular use of aspirin and non-aspirin analgesics (>2 times/week), marital status, total energy intake, personality traits
Simoes Maria et al. (2020)	Lausanne cohort Lc65+ study	M/F	Switzerland	Items adapted from the PSQI	A self-reported nap ≥ 1 time a week	Depression	Self-reported depression diagnosis	Cross-sectional	Range 66–75	2,628	None
Choi et al. (2020)	UK Biobank	M/F	British	Online self-reported questionnaire	A self-reported nap during the day sometimes or usually in the last 4 weeks	Depressive symptoms	A PHQ-9 score ≥ 10	Cohort follow-up time: 6–8 years	Range 18+	100,517	Participant characteristics (sex, age, assessment center), sociodemographic factors (socioeconomic deprivation, employment status, household income, completion of higher education, urbanicity, household size), and physical health factors (BMI and reported physical illness or disability)
Jing et al. (2020)	CHARLS	M/F	China	Self-reported questionnaire	A self-reported nap after lunch of more than 0 h during the past month	Depressive symptoms	A CES-D-10 score ≥ 10	Cohort follow-up time: 2 years	Range 60+	5,108	Age, gender, marital status, education, residency, health status, chronic disease status, BMI, smoking, and drinking status
Xie et al. (2020)	CHARLS	M/F	China	Self-reported questionnaire	A self-reported nap after lunch of more than 0 min in general during the past month	Depressive symptoms	A CES-D-10 score ≥ 10	Cross-sectional	Range 45+	5,746	Age groups, sex, education, marital, region, BMI, waist circumference, smoking, alcohol drinking, nighttime sleep duration, diabetes status, dyslipidemia, high CRP, and hypertension
Lin et al. (2021)	WELL China	M/F	China	Self-reported questionnaire	A self-reported afternoon nap	Depressive symptoms	WHO-5	Cohort follow-up time: 1 year	Mean 56 ± 13; Range 18–80	3,075	Sex, age, education level, marital status, chronic medical history, pre-COVID-19 outbreak BMI, depressed mood status before COVID-19 outbreak, and napping status before COVID-19 outbreak, self-assessment of sleep quality at night during COVID-19 outbreak and sleep duration at night during COVID-19 outbreak, the length of nighttime sleep during the COVID-19 outbreak

The US, the United States; the UK, the United Kingdom; F, female; M, male; BMI, body mass index; GDS, The Geriatric Depression Scale; CIDI-SF, the Chinese version of the computerized Composite International Diagnostic Inventory-short form; PHQ-9, the Patient Health Questionnaire−9; CES-D-10, the 10-item Chinese version of the Center for Epidemiological Studies Depression scale; PSQI: the Pittsburgh Sleep Quality Index; WELL China, Wellness Living Laboratory-China; WHO-5, the World Health Organization-Five Wellbeing Index; HLS, a healthy lifestyle score; SUN, the “Seguimiento Universidad de Navarra” project; CPR, C-reactive protein; SSRI, selective serotonin reuptake inhibitor; MET, the amount of daily physical activity was represented by metabolic equivalent hours per day.

**Table 2:** Quality assessment of included cohort studies.

**Original studies**	**Selection**				**Comparability**	**Outcome**			**Total score**	**Level of quality**
	**(1) Representativeness of the exposed cohort**	**(2) Selection of the non-exposed cohort**	**(3) Ascertainment of exposure**	**(4) Demonstration that the outcome** ** of interest was not present at the start** ** of the study**	**(1) Comparability of cohorts based on the design or analysis**	**(1) Assessment of outcome**	**(2) Was follow-up long enough for outcomes** ** to occur**	**(3) Adequacy of follow-up** ** of cohorts**		
Ruiz-Estigarribia et al. (2019)	1	1	0	1	1	1	1	1	7	High
Choi et al. (2020)	1	1	0	1	1	1	1	0	6	Medium
Jing et al. (2020)	1	1	0	0	1	1	0	1	5	Medium
Lin et al. (2021)	1	1	1	1	1	1	0	1	7	High

**Table 3:** Quality assessment of included cross-sectional studies.

**Original studies**	**(1)**	**(2)**	**3)**	**(4)**	**(5)**	**(6)**	**(7)**	**(8)**	**(9)**	**(10)**	**(11)**	**Total score**	**Level of quality**
Foley et al. (2007)	1	1	1	0	0	1	1	1	0	1	0	7	High
Leblanc et al. (2015)	1	1	1	0	0	1	1	1	0	0	0	6	Medium
Liu et al. (2018)	1	1	1	1	0	1	0	1	0	0	0	6	Medium
Simoes Maria et al. (2020)	1	1	1	1	0	1	1	0	0	1	0	7	High
Xie et al. (2020)	1	1	1	1	0	1	1	1	0	0	0	7	High

(1) Define the source of information (survey, record review).

(2) List inclusion and exclusion criteria for exposed and unexposed subjects (cases and controls) or refer to previous publications.

(3) Indicate the time period used for identifying patients.

(4) Indicate whether or not subjects were consecutive if not population-based.

(5) Indicate if evaluators of subjective components of the study were masked to other aspects of the status of the participants.

(6) Describe any assessments undertaken for quality assurance purposes (e.g., test/retest of primary outcome measurements).

(7) Explain any patient exclusions from the analysis.

(8) Describe how confounding was assessed and/or controlled.

(9) If applicable, explain how missing data were handled in the analysis.

(10) Summarize patient response rates and completeness of data collection.

(11) Clarify what follow-up, if any, was expected and the percentage of patients for which incomplete data or follow-up was obtained.

### Quantitative synthesis

**Figure 2:**
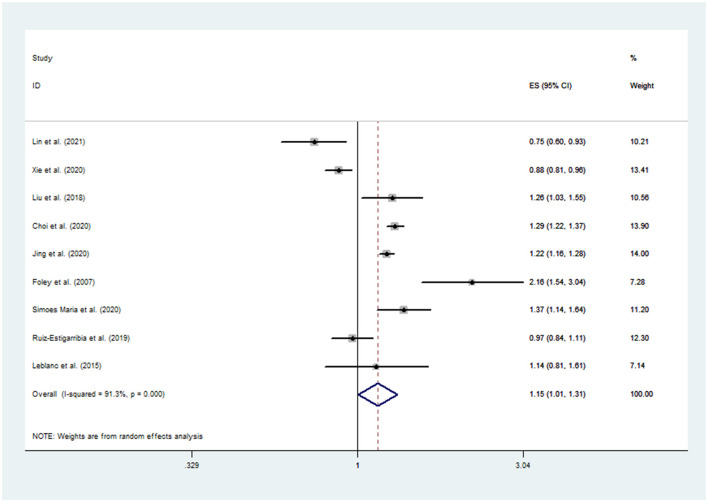
Forest plot of the association between daytime napping and the risk of depression.

Figure 2 shows the pooled results from the random-effects model and the ORs of the included studies. Among the included studies, five reported a positive relationship between daytime naps and the risk of depression (Begg and Mazumdar, 1994; Higgins and Thompson, 2002; Moher et al., 2009; Wallace et al., 2009; Wells et al., 2019). The pooled OR was 1.15 (95% CI: 1.01–1.31). The result showed a positive association between daytime naps and the risk of depression with a high level of heterogeneity (*I*^2^ = 91.3%, *P* < 0.001).

### Subgroup analysis

Subgroup analyses were conducted by study design, daytime nap category, depression measurement, sample size, study quality, study location, drinking status, and sleep duration controlled or absent in the models (Table 4). Due to insufficient data, napping could not be divided by duration or frequency; it was categorized by timing as “afternoon napping” or “napping without a definite timing.” The results of subgroup analyses indicated that the diverse definitions of a daytime nap might be a source of heterogeneity. Napping without a definite timing was significantly associated with an increased risk of depression (OR 1.36, 95% CI: 1.19–1.55; *I*^2^ = 58.3%, *P* = 0.048), and heterogeneity was moderate among these studies (Foley et al., 2007; Leblanc et al., 2015; Liu et al., 2018; Choi et al., 2020; Simoes Maria et al., 2020), while an afternoon nap showed no significant association (OR 0.95, 95% CI: 0.76–1.19), and heterogeneity was high (*I*^2^ = 94.6%, *P* < 0.001) among studies. For the studies during the general period, the risk of napping during depression increased (OR 1.20, 95% CI: 1.06–1.37), while it decreased for those during the special period (OR 0.75, 95% CI: 0.60–0.93). The results from other subgroup analyses showed no significant associations between napping and depression, with a high level of heterogeneity.

**Table 4:** Subgroup analysis of odd ratios for the association between daytime napping and depression.

**Subgroup**	**No of studies**	***OR* (95% CI)**	***I*^2^ (%)**	***P* value for heterogeneity**	***P* value between groups**
**Study location**					
Asia	4	1.01 (0.80, 1.27)	94.4	<0.001	0.334
North America	2	1.57 (0.84, 2.94)	85.1	0.010	
Europe	3	1.20 (0.99, 1.45)	86.5	0.001	
**Daytime napping definition**					
Afternoon nap	4	0.95 (0.76, 1.19)	94.6	<0.001	0.036
Nap without definite timing	5	1.36 (1.19, 1.55)	58.2	0.048	
**Type of depression measure**					
Self-reported scales	6	1.08 (0.92, 1.25)	92.8	<0.001	0.458
Physician diagnosis	3	1.38 (0.94, 2.04)	91.0	<0.001	
**Sample size**					
<10,000	6	1.15 (0.93, 1.42)	93.0	<0.001	0.688
>10,000	3	1.17 (0.96, 1.42)	85.5	0.001	
**Study design**					
Cross-sectional	5	1.28 (0.96, 1.71)	90.9	<0.001	0.483
Cohort	4	1.08 (0.93, 1.24)	90.8	<0.002	
**Study period**					
General period	8	1.20 (1.06, 1.37)	90.7	<0.001	0.067
Special period	1	0.75 (0.60, 0.93)	–	–	
**Controlling drinking**					
Adjusted	3	1.10 (0.86, 1.41)	95.1	<0.001	0.923
Unadjusted	6	1.19 (0.97, 1.46)	89.0	<0.001	
**Controlling sleep duration**					
Adjusted	3	1.94 (0.73, 1.21)	84.9	0.001	0.08
Unadjusted	6	1.25 (1.12, 1.40)	80.6	<0.001	

### Sensitivity analysis

Sensitivity analysis was adopted to identify potential heterogeneity in the association between daytime naps and depression; this helped examine the influence of various exclusions on the combined OR and test the stability of the quantitative synthesis results. The pooled OR ranged from 1.07 (95% CI: 0.91–1.27) to 1.15 (95% CI: 1.00–1.34) when one study was omitted. The leave-one-out analysis indicated that none of the individual studies significantly influenced the overall result.

### Publication bias

**Figure 3:**
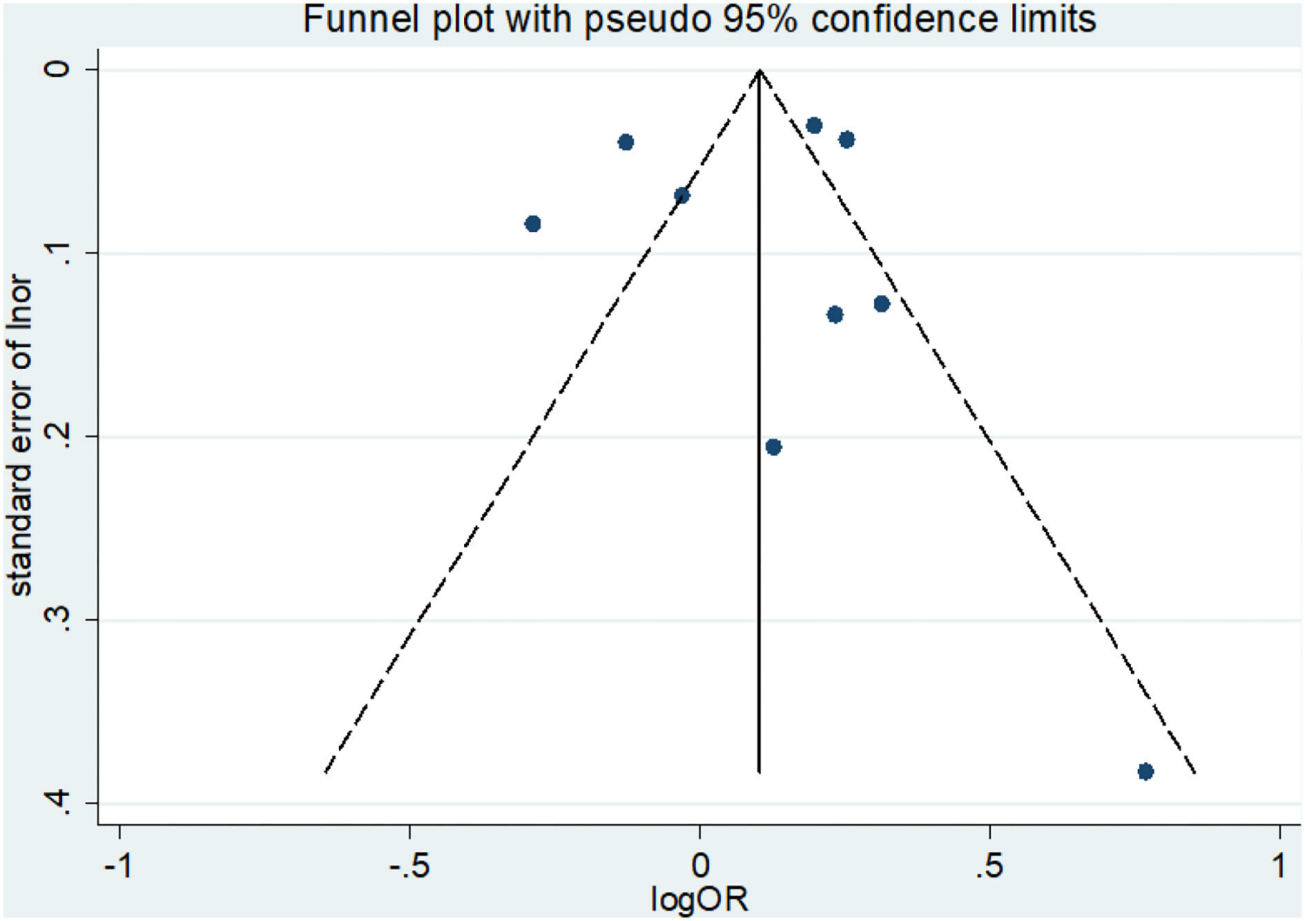
Funnel plot for studies of daytime napping in relation to the risk of depression.

The visual inspection of the funnel plot did not reveal any significant asymmetry (Figure 3). The Egger's and Begg's tests indicated no obvious publication bias among the studies (Egger's test *t* = −0.41, *P* = 0.693; Begg's test *z* = −0.42, *P* = 0.677).

## Discussion

To the best of our knowledge, this study is the first meta-analysis of the association between daytime naps and depression. A growing body of research discovered that only one of the nine studies included had been published before 2014, suggesting growing attention to the effects of daytime naps in recent years. The pooled results (OR = 1.15; 95% CI: 1.01–1.31) indicated that nappers were a little more likely to develop depression than non-nappers based on evidence from the available observational studies.

A possible explanation is that daytime napping may be a result secondary to or a symptom of poor health status and sleep disorders, which could be independent risk factors for depression. Some studies indicated that people with chronic diseases have an increased risk of depression (Ding et al., 2016; Bokenberger et al., 2017; Bouloukaki et al., 2021). Many studies confirmed that sleep disorders increase the risk of depression (Jaussent et al., 2011; Roberts and Duong, 2014; Yu et al., 2016). Previous studies indicated that the association between daytime naps and depression might be attributed to disturbed sleep, such as insomnia, excessive daytime sleepiness, and sleep apnea, which may increase the frequency of naps (LaGrotte et al., 2016; Li et al., 2016). However, daytime napping is also related to the post-lunch dip even after a full night of sleep, especially for habitual nappers (Bes et al., 2009). Some studies suggested that napping was not associated with sleep disorders (Metz and Bunnell, 1990; Foley et al., 2007); it may even be purely appetite driven (Boz et al., 2021). Second, excessive daytime sleepiness has been identified as one of the clinical manifestations of depression, and LaGrotte et al. showed that excessive daytime sleepiness is positively associated with depression. Prolonged napping may not be conducive to recovery from depression (LaGrotte et al., 2016; Li et al., 2017).

On the contrary, a study noted that patients with prolonged napping were more likely to restore mental health than patients without napping habits (Schofield and Khan, 2014). Finally, daytime napping may be a side effect of antidepressant treatment, as antidepressants and sleep deprivation therapy may cause daytime naps (Li et al., 2022). A bidirectional relationship was observed in a 14-year longitudinal study between excessive daytime napping and Alzheimer's disease (Uher et al., 2012) and might also exist between napping and depression.

A study estimated that the prevalence of major depressive disorders and anxiety disorders had increased greatly due to the COVID-19 pandemic (COVID-19 Mental Disorders Collaborators, 2021). However, the subgroup analysis by study period showed that napping was protective against depression during the COVID-19 pandemic; this may be because the behavior of napping indicates a more regular life and work during the COVID-19 pandemic, and these people had a higher level of mental health. Although a high degree of heterogeneity was observed in the meta-analysis, a meta-regression analysis showed that the definition of a daytime nap explained most of the potential heterogeneity. Based on previous studies (Milner and Cote, 2009; Schofield and Khan, 2014), people chose to nap for various reasons, such as appetitive napping for enjoyment, naps in response to sleep loss, and napping in preparation for sleep loss. Milner and Cote (2009) noted that individuals' levels of experience with napping led to different impacts. Daytime naps in people with no experience with daytime naps may be due to fatigue or body aches, which means a poor health condition. These healthier individuals might also be more likely to suffer from depression (Ding et al., 2016; Bokenberger et al., 2017; Bouloukaki et al., 2021). This was consistent with the results of our subgroup analysis studies, which were based on the study location. The risk was highest in North America and lowest in Asia, although none was statistically significant. The age-adjusted prevalence of depression is higher in regions where napping is less popular (such as North America and Australia) and lower in regions where napping is much more popular (such as East Asia and Southern Latin America) (GBD 2019 Mental Disorders Collaborators, 2022). It indicates some complex mechanisms underlying the effects of napping on depression, which should be carefully examined.

The included studies used different measures and criteria to identify depression. In the subgroup analysis by the measure of depression, nappers were likely to be at higher risk for clinical depression than depressive symptoms. However, none of the risks were statistically significant. As self-reported measures of depression are more biased and unreliable compared to physician diagnosis, the risk might be underestimated. A randomized controlled trial showed that self-report and clinician-rated versions of the same instrument uniquely contributed to the prediction of outcome improvement of depression treatment (Uher et al., 2012). Given that the use of clinician-rated scales by psychological physicians in epidemiological studies and routine clinical practice is expensive, self-report scales with a parallel clinician-rated version containing matching content would be a better choice.

Self-reported measures were also widely applied to assess napping, which is common in epidemiological studies. Until now, there have been no valid, standardized measures of daytime napping, which is worse than the lack of valid, standardized measures for the diagnosis of depression. Because all of the included studies used different self-reported questions focusing on different aspects of napping, the identified “nappers” varied greatly across the studies. Some research (Schofield and Khan, 2014; Xie et al., 2020) showed that self-reported naps do not increase the risk of depression. A study noted that objectively measured napping was significantly associated with the risk of depression in very old women (Dautovich et al., 2008). Leng et al. found that subjective napping is uncommon. Self-reported naps may capture only intentional naps, whereas unplanned “snooze” time, which can be captured by actigraphy, is more likely to be missed (Leng et al., 2018). Due to memory biases, self-reported daytime naps are unreliable, and nap details are unlikely to be reported. With the technological development of wearable devices, sleep records can be measured objectively and conveniently, and dose-response effects can be analyzed. Therefore, more studies using objective measures to detect naps are needed so that naps can be determined with definitive and detailed data.

Due to the insufficient information in the original studies, we only grouped the naps in one way, i.e., afternoon naps or naps without definite timing. Subgroup analysis showed that naps without definite timing were significantly associated with an increased risk of depression, while an afternoon nap trended toward an increased risk of depression but was not statistically significant. Opposite results were also obtained: a nap after lunch was a protective factor against depression (Souabni et al., 2022) and cognitive flexibility (Slama et al., 2015). A review in 2021 also found that an early afternoon nap (post-lunch dip time) improved cognitive performance and work efficiency (Dutheil et al., 2021). Evidence from the included studies and other recent studies indicates that the effects of daytime naps might also differ in frequency and duration on depression (Jing et al., 2020; Xie et al., 2020; Lin et al., 2021; Alqurashi et al., 2022), cognitive function (Kitamura et al., 2021), and other health problems (Häusler et al., 2019). Recent large longitudinal (Li et al., 2018) and cross-sectional (Leng et al., 2021) studies suggested the detrimental effects of long naps and the beneficial effects of moderate naps on cognition. Long (usually ≥1 or 1.5 h) and frequent daytime naps may increase the risk of cardiovascular diseases, chronic diseases, and mortality in older people (Zhou et al., 2016; Häusler et al., 2019; Wells et al., 2019; Pan et al., 2020), and people with these diseases are reported to be more likely to have depression. An investigation of the psychological characteristics of males suggested that the differences in sleep needs may be a response to the differences in personality and that long sleepers were worriers; they may have chronic depressive symptoms (Hartmann et al., 1972). These results suggested that different characteristics of naps could have different effects on depression and that naps may be described as a pattern. Moreover, as part of sleep, daytime naps are related to nighttime sleep and the circadian rhythm. Research on many health-related problems focused on the independent and combined effects of nighttime sleep and daytime napping. Jaime et al. found that sleep behavior appeared crucial to further illuminate the health relevance of napping, especially regarding psychological health outcomes (Devine and Wolf, 2016). A large cohort study with 12 years of follow-up by Leng et al. (2019) showed the effects of napping on cognitive impairment differed by nighttime sleep. However, the information provided by the included studies is insufficient to categorize naps in detail and explore the effects of various types of naps on depression and their mechanisms. Napping should be integrated into sleep patterns along with nighttime sleep.

It was observed that the daytime nap effects on health might differ depending on people's age (Liu et al., 2018). A cohort study on people under the average age of 40 found that daytime naps did not increase the risk of depression, although the association was not statistically significant (Ruiz-Estigarribia et al., 2019). Simoes Maria et al. (2020) showed that daytime napping was significantly associated with an increased risk of depression in people over 60. A study on elementary school children showed that napping was significantly associated with improved cognition and fewer emotional/behavioral problems (Liu et al., 2019). These studies showed that napping has different effects on physical conditions in different age groups. Milner suggested that several factors (e.g., age, duration, frequency of the nap, and individual differences in napping experience) may influence the degree of the benefit accrued from a daytime nap (Milner and Cote, 2009). Individuals' sleep needs and body functions change as they age, resulting in different effects of napping on different age groups. Studies have noted that long daytime naps could cause sleep inertia, which has a detrimental effect on people (Leng et al., 2014a; Yamada et al., 2015). However, we did not carry out a subgroup analysis by age because most participants were older adults, and only one included study (Liu et al., 2018) provided age-specific results. The health effects of napping have been studied among young, healthy adults and even athletes (Asplund, 1996; Gillberg et al., 1996; Brooks and Lack, 2006; Faraut et al., 2011). Workplace napping has become popular in more regions and at world-famous companies such as Google, NASA, and Samsung. Further, work can also influence the relationship between napping and depression in many aspects: the availability and schedule (timing, duration, and frequency) of napping, night sleep (e.g., shift work), and income. Further studies should pay more attention to different people.

With the increase in sleep problems and the growing prevalence of depression, preventing mental and physical illnesses associated with sleep disorders has become a widespread public health concern. Napping may be secondary to reduced sleep quality due to poor health, which may be a risk factor for depression. For example, insomnia leads to depression and is accompanied by an increased tendency to nap. Few of the included studies focused on napping or other sleep problems in our meta-analysis (2016) on insomnia and the risk of depression. However, there was a progressive focus on the independent and combined effects of insomnia, length of sleep at night, and napping in a study on sleep and cardiovascular risks. Insomnia or sleep deprivation treatment secondary to depression results in daytime napping and sleepiness. Some cross-sectional studies indicated that, although the current evidence is conflicting, the close relationship between napping and emotional control and cognitive function suggests a substantial relationship between daytime napping and depression.

The underlying biological mechanisms linking daytime naps and the risk of depression are still unknown. Evidence from recent studies revealed that increased inflammation and hyperactivity of the hypothalamic-pituitary-adrenal (HPA) axis have been demonstrated to be two of the most consistent biological findings in major depression (Pariante, 2017). A recent systematic review based on population-based studies found that C reactive protein (CRP), an important inflammatory marker, may be considered a valuable biomarker for major depressive disorder, as most included studies showed higher blood CRP levels were associated with greater symptom severity and a worse response to treatment in patients with depression (Orsolini et al., 2022). Clinical trials also showed the association between CRP and depression severity (Köhler-Forsberg et al., 2017) and improved CRP as a differential predictor of the outcome of depression treatment (Uher et al., 2014). Leng et al. (2014a) found that daytime napping significantly increased CRP levels based on a large population-based cohort study. Further, increased napping is an independent predictor of high CRP by excluding the interactive effects of napping and nocturnal sleep (Mantua and Spencer, 2015). Besides, increased cortisol levels and hyperactivity of the HPA axis induced by glucocorticoid resistance coexist with inflammation in depression patients (Pariante, 2017). Two studies by Woods et al. measured evening cortisol in nappers and found that it was elevated, especially in those with unstable (Woods and Yefimova, 2012) and longer (Woods et al., 2013) napping episodes. All these lines of evidence indicated the existence of molecular and clinical mechanisms underlying or shared by daytime napping and depressive disorders.

Studies over the last few years have demonstrated that depression and mental health problems are no longer seen only as disorders of the mind or the brain; they are a disease of the whole body. Depression is hereditary, and a number of genetic variants associated with depression have been identified from genome-wide association studies (Mullins and Lewis, 2017). There is also a genetic component to daytime napping behavior. A recent study by researchers from different countries identified genetic variants associated with daytime naps and three potential mechanisms that promote napping. This study further provided preliminary evidence on potential causal links between more frequent daytime naps and higher blood pressure and waist circumference (Dashti et al., 2021). Genome-wide association analysis was used in one of the included studies. The results showed napping as the top risk factor among the 29 identified risks or protective factors significantly associated with depression (Choi et al., 2020). In the future, similar studies should be carried out to elucidate genetic links between napping and depressive disorders and provide personalized recommendations for napping.

## Strengths and limitations

The meta-analysis in this study highlighted the synthesized effects of daytime napping on the risk of depression for the first time in an international public publication. First, it included a total of 648,711 participants from six countries in Europe, Asia, and North America. The large sample size and broad geographic coverage significantly increased the statistical power and generalizability of the associations between daytime napping and depression risks. Second, four cohort studies were included, which could provide longitudinal evidence demonstrating temporal relationships between daytime naps and the risk of depression. Third, all the included studies were of moderate or high quality, with more convincing results.

Some potential limitations in this study should also be discussed. First, five of the nine original studies included were cross-sectional studies. Only longitudinal studies can demonstrate the effect of a risk factor (i.e., daytime naps) on health conditions (i.e., depression). Second, the original study information was insufficient to distinguish between the definition and types of napping. According to available studies, different types of naps could have different relationships with or effects on health status. Thus, the summary could not be independently made about the relationship between various types of naps and depression. Third, a spectrum of depressive disorders and napping were included. The use of self-reported questionnaires rather than physician diagnosis or actigraphy to examine the relationship between daytime naps and depression is bound to introduce recall bias and measurement heterogeneity. It would be feasible to measure naps using objective instruments with wearable devices in large epidemiological studies in the future. Fourth, although each included study had various adjusted confounding factors, some important confounding factors (e.g., other health problems and sleep quality) were not fully adjusted, which might influence the association between daytime naps and depressive risk. Therefore, the results in this study were extracted with fully adjusted covariates.

Based on the meta-analysis results, this study found few longitudinal studies on naps and depression. Future studies should focus more on the causal and temporal relationships between naps and depression. In addition, researchers should pay special attention to the duration, timing, and frequency of naps and explore their effects on depression with likely distinct biological mechanisms.

## Conclusions

In summary, the meta-analysis indicates that daytime napping is likely to increase the risk of depression. Given the high heterogeneity, various methods, and conflicting evidence, the results should be viewed critically. The effects of daytime napping on depression may vary largely, and the detriments or benefits of napping differ depending on people's characteristics (e.g., age and geographic region), the napping pattern (e.g., duration, frequency, timing), and sleep experience. To specify the effects, objective measures of napping, usually using actigraphy with wearable devices and valid self-report scales for depression with a parallel clinician-rated version containing matching content, were a priority in epidemiological studies. The underlying mechanisms between daytime naps and the risk of depression are still unclear; however, genetics, inflammation, and hyperactivity of the HPA axis may play an important role. Future studies are required to explore the causal relationship and underlying biological mechanisms between daytime napping and depression to gain a better understanding of the relationship. With depression becoming more common and daytime naps becoming more popular in modern society, these findings have significant implications for future studies on depression.

## Data availability statement

The raw data supporting the conclusions of this article will be made available by the authors, without undue reservation.

## Author contributions

CW and LL conceived the study. GZ, HH, JZ, XK, SY, DY, and ZC were responsible for collecting and cleaning the data and providing assistance with writing the manuscript. LL and QZ wrote the manuscript. LZ, YG, and ZL contributed to the review and revision of the study. CW is the guarantor of this work and, as such, has full access to all the data in the study and takes responsibility for the integrity of the data and the accuracy of the data analysis. All authors contributed to the article and approved the submitted version.
